# Aorta Dilatation in Unrepaired Tetralogy of Fallot

**DOI:** 10.7759/cureus.49212

**Published:** 2023-11-21

**Authors:** Long Lu, Qingxu Guo, Zhentian Cui

**Affiliations:** 1 Department of Cardiovascular Surgery, Seventh Medical Center, Chinese People’s Liberation Army (PLA) General Hospital, Beijing, CHN

**Keywords:** aortic arch, ascending aorta, aortic sinotubular junction, aortic annular, tetralogy of fallot, aortic root dilation

## Abstract

Background

Aortic root dilation is an increasingly recognized feature in repaired tetralogy of Fallot (TOF) patients. However, the dilation at the aortic root and ascending aorta in unrepaired TOF is rarely studied. This study aims to confirm whether aortic dilation is a common feature in unrepaired TOF and investigate the factors attributed to aortic dilation.

Methodology

Patients with an echocardiographic diagnosis of TOF undergoing computed tomography angiography were retrospectively studied. Diameters and z scores of aortic annular (Ao1), aortic sinotubular junction (Ao2), ascending aorta (Ao3), and distal transverse aortic arch (Ao4) were measured. Preoperative body surface area (BSA), hemoglobin (Hb), hematocrit (HCT), arterial oxygen saturation (SaO_2_), and platelet (PLT) count were recorded.

Results

A total of 101 TOF patients aged 6.8 ± 9.5 years were included in this study, whose mean BSA (m^2^), Hb (g/L), HCT, SaO_2_ (%), and PLT (10^9^/L) were 0.7 ± 0.4, 162.1 ± 3.8, 0.5 ± 0.1, 85.1 ± 9.3, and 238.1 ± 101.1, respectively. The mean z score of Ao1, Ao2, Ao3, and Ao4 were 10.3 ± 3.5, 4.7 ± 2.9, 4.0 ± 2.7, and 4.1 ± 2.4, respectively. Age and BSA were positively correlated with the z scores of Ao1 and Ao2. Preoperative Hb and HCT were positively correlated with the z scores of Ao1, Ao2, Ao3, and Ao4. Preoperative SaO_2_ and PLT were negatively correlated with the z scores of Ao1, Ao2, Ao3, and Ao4.

Conclusions

Aortic dilation is common in unrepaired TOF patients. The dilation of different levels of the aorta was correlated with age, BSA, preoperative Hb, HCT, SaO_2_, and PLT.

## Introduction

Tetralogy of Fallot (TOF) is the most common cyanotic congenital heart disease. Early detection of TOF leads to prompt surgery, contributing to improvements in long-term prognosis and overall survival.

Aortic dilatation after repaired TOF (rTOF) has been widely reported and regarded as an adverse event in rTOF patients [1,2]. Male gender, late TOF repair, prior palliative shunt, and longer intervals of shunt-to-surgical repair are predisposing factors for aortic dilatation after surgery [3-5]. Aortic dilatation has also been reported in fetal TOF [6,7]. Intrinsic histological abnormalities of the aortic wall have been noted among fetuses [8], which suggests that aortic dilation started even before birth.

Although aortic valve regurgitation and dilated aorta in unrepaired TOF patients have not been noted clinically, little research has addressed this fact. Thus, this study aimed to confirm whether aortic dilation is common in unrepaired TOF and investigate the factors correlating to aortic dilation.

## Materials and methods

Study population

This study was approved by our institutional review board (approval number: CPGH-2021265). All procedures were performed in accordance with our institutional guidelines to protect patients’ confidentiality. Informed consent was not required owing to the retrospective nature of this study.

Patients with an echocardiographic diagnosis of TOF undergoing computed tomography angiography (CTA) during the study period were included. Patients with palliative shunt or repair were excluded. Patients affected by active or chronic inflammatory or autoimmune diseases, those with active or past hematological proliferative diseases or oncological history, those who received blood transfusions, those who were treated with anti-inflammatory drugs in the last three months, or those with active infection were excluded. Blood sampling was done from the antecubital vein at admission [9]. Blood samples were immediately sent to the laboratory for analysis. Ethylenediamine-tetraacetic acid-containing tubes were used for the hemogram assessment. A complete blood count test, including differentials, was evaluated using an automated blood cell counter [9]. A total of 101 patients (62 males and 39 females) from 2014 to 2018, with a median age of 6.8 years (range = 0.3-45.8 years) and a median weight of 18.6 kg (range = 5-82 kg), were included.

Data collection

We collected patients’ demographic data, perioperative clinical information, echocardiographic data, and CT data for analysis. In cases of TOF, a CT scan is typically performed to assess the range and size of the stenotic portion of the pulmonary artery before surgery, particularly when echocardiography indicates pulmonary artery stenosis. Helical CT was performed on a single-detector scanner (256-slice, GE Revolution Healthcare). One dose of 1.5 mL/kg (maximal of 80 ml) non-ionic, low-osmolar contrast agent (Iomeprol 400; Patheon Italia S.P.A.) with a concentration of 300 mg/ml was used for pediatric patients. The images were reconstructed onto the mediastinum using the zoom feature with a field of view of 220 mm and a matrix of 512 × 512. The region of interest involving the aorta and its main branches was outlined [10]. The widths of Ao1, Ao2, Ao3, and Ao4 were measured at the transverse section of the aortic annular, sinotubular junction, ascending aorta, and distal aortic arch. The diameter of Ao1, Ao2, Ao3, and Ao4 was measured using one of the most recent imaging modalities of helical CT. The measurements were evaluated by two blinded experienced readers and decisions were made in consensus. Other parameters such as the diameter of the main PA and the laterality of the aortic arch were also recorded. Preoperative hemoglobin (Hb), hematocrit (HCT), SaO_2_, and platelet (PLT) count were collected from the medical records.

Statistical analysis

SPSS version 22.0 (IBM Corp., Chicago, IL, USA) was used for data analysis. Continuous data were presented as mean and standard deviation (SD). Categorical variables were presented as proportions. Pearson’s correlation was used to determine the correlation between z scores of aortic dimension and age, body surface area (BSA), Hb, HCT, SaO_2_, and PLT. A p-value <0.05 was considered statistically significant.

## Results

Aortic dilation

**Figure 1:**
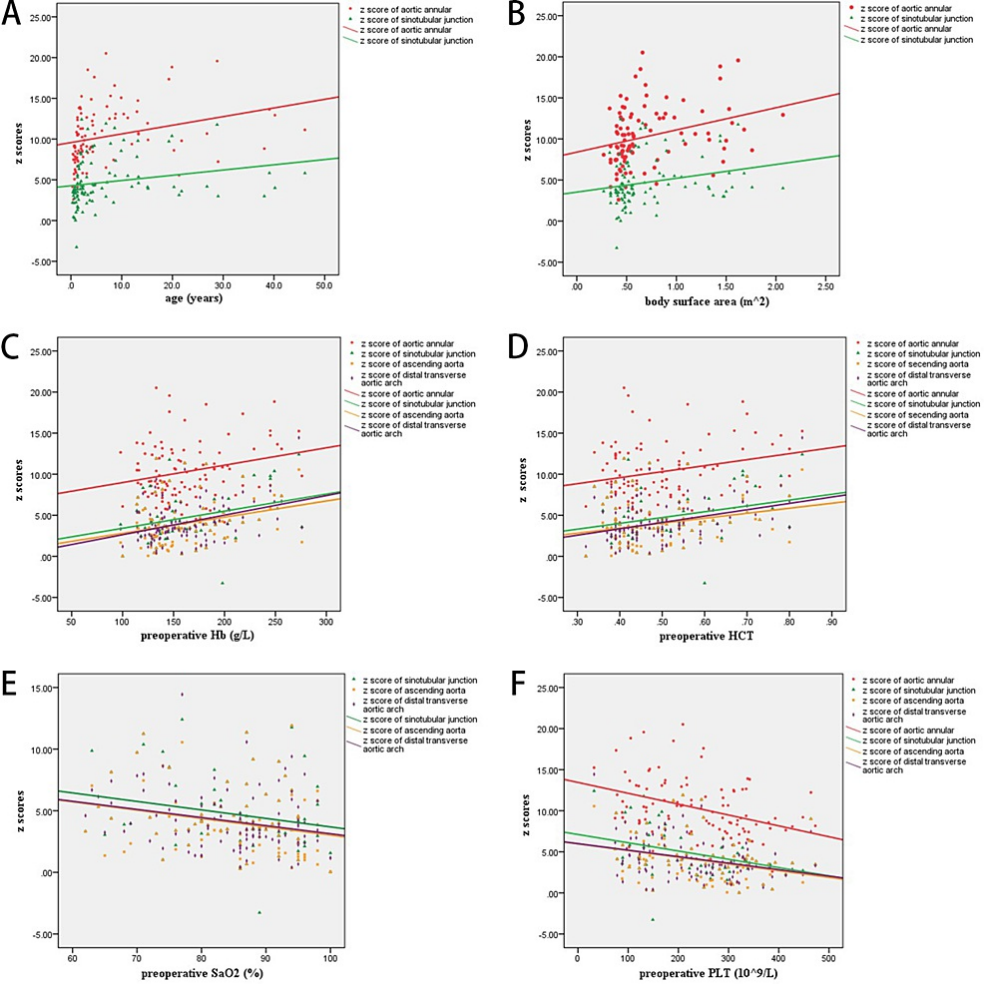
Age and BSA were positively correlated with the z scores of Ao1 and Ao2 (A-B). Preoperative Hb and HCT were positively correlated with the z scores of Ao1, Ao2, Ao3, and Ao4 (C-D). Preoperative SaO2 and PLT were negatively correlated with the z scores of Ao1, Ao2, Ao3, and Ao4 (E-F). Ao1: aortic annular; Ao2: aortic sinotubular junction; Ao3: ascending aorta; Ao4: distal transverse aortic arch; BSA: body surface area; SaO_2_: arterial oxygen saturation; Hb: hemoglobin; PLT: platelet; HCT: hematocrit

Table 1 demonstrates patient demographics and the z scores of Ao1, Ao2, Ao3, Ao4, which were 10.3 ± 3.5, 4.7 ± 2.9, 4.0 ± 2.7, and 4.1 ± 2.4, respectively. Table 2 presents the correlation between the z scores of the aorta at the four levels and demographic data. Age and BSA were positively correlated with the z scores of Ao1 and Ao2 (Figures 1A, 1B). Preoperative Hb and HCT were positively correlated with the z scores of Ao1, Ao2, Ao3, and Ao4 (Figures 1C, 1D). Preoperative SaO_2_ and PLT were negatively correlated with the z scores of Ao1, Ao2, Ao3, and Ao4 (Figures 1E, 1F).

**Table 1:** Patient characteristics. Ao1: aortic annular; Ao2: aortic sinotubular junction; Ao3: ascending aorta; Ao4: distal transverse aortic arch; SaO_2_: arterial oxygen saturation; Hb: hemoglobin; PLT: platelet; HCT: hematocrit

	N = 101
Age (years)	6.8 ± 9.5
Female gender (%)	39 (38.6%)
Body weight (kg)	18.6 ± 15.8
body surface area (m^2^)	0.7 ± 0.4
z score of Ao1	10.3 ± 3.5
z score of Ao2	4.7 ± 2.9
z score of Ao3	4.0 ± 2.7
z score of Ao4	4.1 ± 2.4
SaO_2_ (%)	85.1 ± 9.3
Hb (g/l)	162.1 ± 3.8
PLT (10^9^/L)	233.8 ± 101.1
HCT (%)	0.5 ± 0.1

**Table 2:** Correlation between z scores of aortic dimension and demographics in patients with TOF. *: P-value <0.05 was considered statistically significant. Ao1: aortic annular; Ao2: aortic sinotubular junction; Ao3: ascending aorta; Ao4: distal transverse aortic arch; BSA: body surface area; SaO_2_: arterial oxygen saturation; Hb: hemoglobin; PLT: platelet; HCT: hematocrit

Variables	Ao1		Ao2		Ao3		Ao4	
	r	P-value	r	P-value	r	P-value	r	P-value
Age	0.286	0.004*	0.214	0.032*	0.141	0.161	0.118	0.241
BSA	0.300	0.002*	0.231	0.020*	0.162	0.106	0.106	0.292
Hb	0.233	0.019*	0.282	0.004*	0.287	0.004*	0.392	<0.001*
HCT	0.239	0.016*	0.285	0.004*	0.263	0.008*	0.379	<0.001*
SaO_2_	-0.128	0.202	-0.224	0.024*	-0.236	0.017*	-0.260	0.009*
PLT	-0.377	<0.001*	-0.354	<0.001*	-0.309	0.002*	-0.337	0.001*

Correlation between PLT and preoperative Hb and SaO_2_

**Figure 2:**
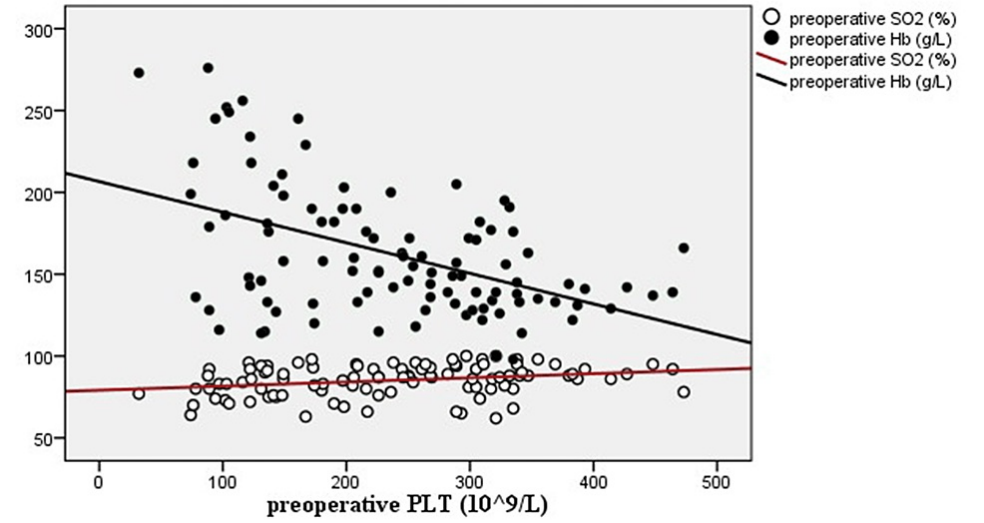
Hb was negatively and SaO2 was positively correlated with PLT. Hb: hemoglobin; PLT: platelet

Table 3 exhibits the correlation between preoperative PLT and preoperative Hb and SaO_2_. Hb was negatively, and SaO_2_ was positively correlated with PLT. The tendency is directly shown in Figure 2.

**Table 3:** Correlation between PLT count and Hb concentration and SaO2. SaO_2_: arterial oxygen saturation; Hb: hemoglobin; PLT: platelet

Variable	Hb concentration		SaO_2_	
	r	P-value	r	P-value
PLT count	-0.485	<0.001	0.274	0.006

## Discussion

This study comprehensively demonstrates the high prevalence of aortic dilation in unrepaired TOF patients. This highlights that aortic dilation occurred after surgical repair for TOF [1,2]. The dilation occurs at different levels including the aortic annular, sinotubular junction, ascending aorta, and aortic arch. Our data suggested that the dilation of the aortic annular was more serious than the other three levels. The z scores of aortic annular and sinotubular junction were positively correlated with age and BSA, which is partly due to the increased aortic flow resulting from right to left shunt, further implicating the progressive dilation of the aorta disproportionate to body growth [3]. Persistent right ventricle outlet obstruction before TOF repair leads to blood deviation to the left heart and aorta, with aortic overflow working as a facilitating factor for aortic annular and sinotubular junction dilation. As many studies have reported, aortic root dilation would proceed even after surgical repair for TOF, with the underlying mechanism of aortic dilation likely being the histological abnormalities of the aorta [5-8]. What we have known about histological abnormalities so far is the fragmentation or loss of elastic fibers and the loss of smooth muscle cells. These histological changes are genetically programmed and will last after surgical repair for TOF.

Another important finding is the positive correlation between aortic dilation and preoperative Hb, as well as between aortic dilation and HCT. It was reported more than two decades ago that increased Hb is related to the increase in systemic resistance [11-13]. Increased aortic pressure leads to aortic dilation gradually. The increase in systemic resistance is not a result of vascular resistance alteration but of the increase in blood viscosity [14-17]. Although higher Hb aggravates the risk of aortic dilation and systemic stroke, it is also helpful in reducing right to left shunt and promoting an increase in pulmonary blood flow in TOF patients. From this perspective, increased Hb and HCT work as a beneficiary mechanism in TOF for improving arterial oxygenation.

Thus, it is easy to understand the correlation between aortic dilation and preoperative SaO_2_. The lower the preoperative SaO_2 _and the higher Hb, the more likely the aorta is to dilate. On the other hand, lower SaO_2_ indicates more right to left blood shunt and higher volume overload of the aorta. Furthermore, the direct positive correlation between aortic dilation and aortic blood flow has been proven [9,12], which is the reason why aortic dilation is negatively correlated with preoperative SaO_2_. To our knowledge, this is the first study to determine the significant correlation between aortic dilation and preoperative Hb, HCT, and SaO_2_ in unrepaired TOF patients.

Interestingly, among the set of unrepaired TOF in this study, the preoperative PLT was negatively correlated with the z scores of aortic dimension, although the underlying reason remains unclear. Olgun et al. demonstrated that PLT activation was increased in patients with cyanotic congenital heart disease [18]. According to our analysis, preoperative Hb was negatively and SaO2 was positively correlated with PLT, highlighting the negative correlation between z scores of aortic dimension and PLT [19,20].

Considered together, our results showed more significant aortic dilation, lower SaO_2_, higher Hb, and less PLT. This pathophysiological change occurred in unrepaired TOF patients, which could not only enhance the ability of blood to transport oxygen but also reduce the risk of thromboembolic complications.

Limitations and recommendations

This is a cross-sectional, retrospective, and single-center study. As the size of the sample was not large enough and there were fewer adult patients, we could not stratify this set of patients according to age, which could have resulted in more convincing findings. Medical factors affecting aortic dilatation such as hypertension, collagen diseases, gestational history, and smoking were not identified.

## Conclusions

Aortic dilation is common in unrepaired TOF. The dilation can occur from the aortic annular to the aortic arch, with aortic annular dilation being the most serious. Age, BSA, preoperative Hb, HCT, SaO_2_, and PLT are all correlated with aortic dilation.
